# Effects of Early Changes in Blood Pressure During Intravenous Thrombolysis on the Prognosis of Acute Ischemic Stroke Patients

**DOI:** 10.3389/fnagi.2020.601471

**Published:** 2020-12-04

**Authors:** Zhong-Xiu Wang, Chao Wang, Peng Zhang, Yang Qu, Zhen-Ni Guo, Yi Yang

**Affiliations:** ^1^Department of Neurology, Stroke Center and Clinical Trial and Research Center for Stroke, The First Hospital of Jilin University, Changchun, China; ^2^China National Comprehensive Stroke Center, Changchun, China; ^3^Jilin Provincial Key Laboratory of Cerebrovascular Disease, Changchun, China

**Keywords:** acute cerebral infarction, blood pressure variability, intravenous thrombolysis, outcome, spontaneous blood pressure decrease

## Abstract

**Background**: Intravenous thrombolysis (IVT) therapy is currently one of the best medical treatments available for patients with acute ischemic stroke. Studies have shown that blood pressure (BP) changes in patients treated with IVT are significantly correlated with prognosis.

**Objective**: Our study aimed to determine the relationship between BP changes during recombinant tissue plasminogen activator (rt-PA) infusion and the 3-month prognosis evaluated using the modified Rankin Scale (mRS) and determine the factors influencing BP changes during rt-PA infusion.

**Methods**: Consecutive patients who were treated with IVT and admitted to our stroke center between May 2015 and October 2017 were analyzed retrospectively. Patients were divided into two groups according to their 3-month prognosis status: patients with mRS ≤ 2 were defined as “favorable outcome group” and those with mRS ≥ 3 as “unfavorable outcome group”. First, the factors affecting prognosis after thrombolysis were analyzed. Second, we analyzed the relationship between BP and the prognosis. BP was taken before and at regular intervals of 15 min during the rt-PA infusion (1 h). The average value of BP during thrombolysis was calculated and compared to the baseline BP. BP decrease was defined as the difference between the baseline BP and the average BP, provided it was greater than 0 mmHg. Third, univariate and multivariate analyses were performed to identify factors that may contribute to BP decrease.

**Results**: In total, 458 patients were included. Patients with a lower baseline National Institute of Health Stroke Scale (NIHSS) score (8.25 ± 5.57 vs. 13.51 ± 7.42, *P* < 0.001), a higher Alberta Stroke Program Early CT Score (ASPECTS; 8.65 ± 1.82 vs. 8.13 ± 2.00, *P* = 0.005), decreased BP during thrombolysis (69.4% vs. 59.8%, *P* = 0.037), and steady BP (SD < 10 mmHg) were more likely to have a favorable outcome (73.9% vs. 60.6%, *P* = 0.019). High baseline BP (OR > 1), hypertension history (OR < 1), and baseline ASPECTS (OR > 1) were independent factors of BP change during thrombolysis.

**Conclusion**: Patients with decreased or steady BP during thrombolysis were more likely to have a favorable outcome. Baseline ASPECTS, baseline NIHSS score, and hypertension history influenced BP changes during thrombolysis.

## Introduction

Stroke is the leading cause of death in the Chinese population because of its high rate of morbidity, disability, mortality, and recurrence, and it negatively affects the national health and quality of life (Wang et al., 2017). Cerebral infarction is the most common type of stroke (Liesch, 2012). Intravenous thrombolysis (IVT) therapy is currently one of the best medical treatments available for patients with acute ischemic stroke. However, individual differences in the effects of IVT therapy are significant. Identifying relevant factors influencing IVT prognosis and conducting targeted interventions are important to improvement in IVT prognosis.

A sudden elevated blood pressure (BP) response to acute ischemic stroke has a high rate of incidence, certain self-limitations, and an effect on prognosis (Aslanyan et al., 2003). A study (Qureshi et al., 2007) of a nationally representative large dataset in the U.S. revealed that elevated BP was observed in about 60% of acute ischemic stroke patients admitted to the emergency department. Some studies (Kellert et al., 2012; Endo et al., 2013) showed that BP variability is relevant to poor outcomes. BP changes, including variability, are closely correlated with cerebral infarction prognosis (Miao et al., 2006; Tikhonoff et al., 2009). However, it is worth noting that the influence of BP fluctuation on stroke patients receiving IVT therapy is still controversial. One study (Aslanyan et al., 2003) has shown that high BP before thrombolysis and significant BP fluctuations after IVT therapy are risk factors for a poor prognosis, whereas another study (Liu et al., 2016) indicated that spontaneous BP decrease within a certain range is due to recanalization and reperfusion of the brain tissue, which is considered good prognostic factors. Thus, the relationship between BP changes and IVT therapy needs further research.

Our study aimed to investigate the relationship between BP changes during recombinant tissue plasminogen activator (rt-PA) infusion and 3-month prognosis evaluated using the modified Rankin Scale (mRS) and determine the factors influencing BP during the infusion to better understand this disease and improve its clinical management.

## Materials and Methods

### Participants

Patients who were treated with IVT and admitted to the stroke center of the First Hospital of Jilin University from May 2015 to October 2017 were analyzed retrospectively. IVT was performed within 4.5 h from onset according to current guideline recommendations and the physician’s decision (Jauch et al., 2013; Powers et al., 2018). After IVT, all patients received standard medical treatment and general care in the stroke center. Patients were excluded from this study if: (1) their laboratory data and/or follow-up data were unavailable; (2) they used intravenous antihypertensive drugs before or during thrombolysis; or (3) they accepted endovascular treatment.

We collected patient data including demographics, medical history, personal and family history, lab test results, imaging, treatment time point, and other related information. Medical history was defined as per the article (Wang et al., 2011) “The China National Stroke Registry for patients with acute cerebrovascular events: design, rationale, and baseline patient characteristics.” Blood lipids were collected in the next morning after IVT.

Brachial BP was measured using an automatic cuff sphygmomanometer before and at regular intervals of 15 min during the infusion of rt-PA (1 h). Because several previous studies have demonstrated that there is a stronger predictive function of systolic BP (SBP) than diastolic BP in patients with ischemic stroke (Tikhonoff et al., 2009; Endo et al., 2013), we collected SBP to evaluate early BP changes. All the BP refers to SBP in the present study. The average value of BP during thrombolysis was calculated and compared to the baseline BP. BP decrease was defined as the difference between the baseline BP and the average BP, provided it was greater than 0 mmHg. BP fluctuation during thrombolysis was measured by the standard deviation (SD), which is the arithmetic square root of variance (Liu et al., 2015).

Baseline Alberta Stroke Program Early CT Score (ASPECTS) was evaluated using brain computed tomography (CT). CT examinations were repeated 24 h after thrombolysis. Hemorrhage transformation was evaluated using the 24 h CT scans, following the Second European Cooperative Acute Stroke Study (ECASS-II) classification.

mRS was evaluated at the 3-month double-blind follow-up after IVT. Doctors who had recorded the cases were not assigned to follow-up. Detailed addresses and phone numbers were documented on admission to the hospital. Patients with mRS ≤ 2 were included in the “favorable outcome group” and those with mRS ≥ 3 into the “unfavorable outcome group.”

### Statistical Analysis

The statistical program for social sciences version 20.0 (SPSS, IBM, West Grove, PA, USA) was used to analyze all data. Continuous variables were expressed as mean (± SD) and were analyzed using a *t*-test. Classified variables were analyzed using the Pearson chi-square test. *P* < 0.05 was considered statistically significant. A bivariate logistic regression model was used to verify independent 3-month prognostic factors of cerebral infarction and factors influencing BP. Variables with *P* < 0.1 were included in the multivariate analysis model. Also, a history of atrial fibrillation, coronary heart disease, and hypertension may be potentially correlated with acute BP changes; thus, they were added to the above model.

## Results

In total, 488 consecutive patients diagnosed with acute ischemic stroke who underwent IVT were screened. Thirty patients who did not meet BP standards or whose laboratory or follow-up data were unavailable were excluded from this study. Finally, 458 patients were included in the study.

### Relationship Between Baseline Data and Cerebral Infarction Prognosis

Baseline National Institute of Health Stroke Scale (NIHSS) score (8.25 ± 5.57 vs. 13.51 ± 7.42, *P* < 0.001) and HDL (1.24 ± 0.32 vs. 1.36 ± 0.41, *P* = 0.012) were significantly lower, whereas the CT ASPECTS (8.65 ± 1.82 vs. 8.13 ± 2.00, *P* = 0.005) was higher in the favorable outcomes group. Additionally, patients with favorable outcomes were more likely to have decreased BP during thrombolysis (69.4% vs. 59.8%, *P* = 0.037). The history of hypertension was significant (40% vs. 54%, *P* = 0.011) in the unfavorable outcome group. Rates of hemorrhage transformation after IVT were significantly different between the two groups (16% vs. 23.9%, *P* = 0.034). BP decrease developed less hemorrhage transformation (OR 0.459, 95% CI 0.276–0.761, *P* < 0.001). Other factors, such as sex, age, cerebral infarction/TIA history, diabetes mellitus, and other common risk factors, did not show a significant effect on prognosis (Supplementary Table 1).

### Relationship Between BP Changes and Prognosis

**Figure 1:**
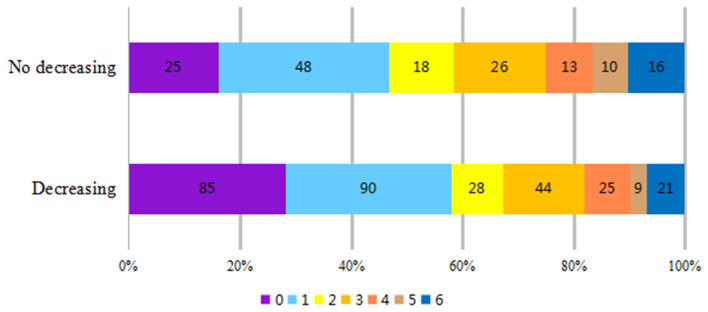
Distribution of 3 m modified Rankin Scale (mRS) after thrombolysis.

Patients were divided into decreasing and no decreasing groups (regardless of fluctuation extent) according to the features of BP during the rt-PA infusion process. There was no significant difference in baseline mRS score (3.62 ± 0.96 vs. 3.79 ± 0.82, *P* = 0.08) between the two groups. The 3-month mRS score was lower in the BP decreasing group (1.82 ± 1.79 vs. 2.62 ± 2.06, *P* = 0.001). Simultaneously, the improvement was more significant (1.79 ± 1.78 vs. 1.17 ± 2.03, *P* = 0.01) and the rate of favorable outcome (mRS ≤ 2) was higher (67.5% vs. 57.7%, *P* = 0.037) in the BP decreasing group. There was no significant difference in fatality rates between the two groups (7.3% vs. 11.5%, *P* = 0.126; Table 1). The number of patients with 3-month mRS ≤ 1 and mRS ≤ 2, which indicates a better prognosis, was significantly higher in the BP decreasing group (Figure 1).

**Table 1:** Relationship between blood pressure (BP) changes and prognosis.

		BP change		
mRS score		Decreasing	No decreasing	*P*
		*n* = 302	*n* = 156	
Baseline mRS		3.62 ± 0.96	3.79 ± 0.82	0.08
3 m mRS		1.82 ± 1.79	2.62 ± 2.06	0.001*
Variation of mRS**		1.79	1.17	0.01*
3 m mRS ≤ 2	*n* (%)	203 (67.5)	91 (57.7)	0.037*
3 m mRS = 6 (death)	*n* (%)	22 (7.3)	18 (11.5)	0.126

*Denotes *P* < 0.05 for comparing between two groups; **denotes value calculated by baseline modified Rankin Scale (mRS) score minus 3 m mRS score.

### Relationship Between BP Fluctuation During Thrombolysis and Prognosis

There was a significant difference in the SD of BP between the BP decreasing group and the no decreasing group (74.8% vs. 62.8%, *P* = 0.007). For the BP decreasing group, the better prognosis was correlated with BP SD < 10 mmHg (73.9% vs. 60.6%, *P* = 0.019) but not with the range of decrease (*P* = 0.235 and *P* = 0.705). For the BP no decreasing group, poor prognosis was correlated with large fluctuations in BP (SD > 10 mmHg; *t*-test: SD: 7.24 ± 4.61 mmHg vs. 9.09 ± 5.97 mmHg, *P* = 0.023; Chi-square test: 76.9% vs. 66.2%, *P* = 0.108; Table 2).

**Table 2:** Relationship between BP fluctuation during thrombolysis and 3 m favorable outcomes.

BP fluctuation during IVT		Prognosis (mRS)		*P*
		mRS ≤ 2	mRS ≥ 3	
**All patients**				
BP standard deviation (SD)	mmHg	8.42 ± 8.77	9.19 ± 6.78	0.325
SD < 10 mmHg	*n* (%)	220 (74.8)	103 (62.8)	0.007*
**BP decreasing**				
BP standard deviation (SD)	mmHg	8.95 ± 10.04	9.26 ± 7.29	0.822
SD < 10 mmHg	*n* (%)	150 (73.9)	60 (60.6)	0.019*
Decrease < 10 mmHg	*n* (%)	98 (48.3)	55 (55.6)	0.235
Decrease < 20 mmHg	*n* (%)	156 (76.8)	78 (78.8)	0.705
**BP no decreasing**			
BP standard deviation (SD)	mmHg	7.24 ± 4.61	9.09 ± 5.97	0.023*
SD < 10 mmHg	*n* (%)	70 (76.9)	43 (66.2)	0.108
Increase < 10 mmHg	*n* (%)	62 (68.1)	39 (60.0)	0.252
Increase < 20 mmHg	*n* (%)	86 (94.5)	52 (80.0)	0.002*

*Denotes *P* < 0.05 for comparing between two groups.

### Factors Influencing BP Decrease During Infusion of rt-PA

Univariate analysis of BP changes involved age, baseline BP, baseline NIHSS score, baseline ASPECTS, and medical history. Univariate analysis showed that patients with a higher baseline BP (154.65 ± 20.49 vs. 146.67 ± 19.57, *P* < 0.001) and a history of hypertension (32.8% vs. 42.3%, *P* = 0.044) were more likely to have a decrease in BP during thrombolysis. Baseline CT ASPECTS also significantly influenced BP (8.30 ± 1.70 vs. 7.88 ± 2.23, *P* = 0.026).

Variables of univariate analysis with statistical significance (*P* < 0.1) and diseases that may affect BP were included in the multivariate analysis. It was noted that baseline BP, history of hypertension, baseline ASPECTS were all independent factors associated with the changes in BP. Patients whose baseline BP (OR 1.02, 95% CI 1.01–1.031, *P* < 0.001) and baseline CT ASPECTS (OR 1.139, 95% CI 1.024–1.267, *P* = 0.017) were higher were more likely to have a decrease in BP during thrombolysis. Additionally, the presence of hypertension also increased the chances of BP decrease during thrombolysis (OR 0.602, 95% CI 0.38–0.952, *P* = 0.030; Table 3).

**Table 3:** Multivariate analysis of BP decrease during recombinant tissue plasminogen activator (rt-PA) infusion.

Factor	*P*	OR	95% CI for OR	
			Lower bound	Upper bound
Baseline CT ASPECT score	0.017*	1.139	1.024	1.267
Baseline BP	<0.001*	1.02	1.01	1.031
Coronary artery disease	0.854	0.935	0.456	1.918
Atrial fibrillation	0.708	1.146	0.561	2.34
History cerebral infarction/TIA history	0.376	1.332	0.706	2.51
Hypertension	0.030*	0.602	0.38	0.953
Diabetes mellitus	0.771	1.095	0.593	2.022

*Denotes *P* < 0.05 for comparing between two groups.

## Discussion

In this study, we examined the effects of early changes in BP during IVT on the prognosis of acute ischemic stroke patients. Patients with a decreased and steady BP during thrombolysis were more likely to have a favorable outcome. Baseline BP, hypertension history, and baseline CT ASPECTS were found to be factors influencing the BP change.

The relationship between BP changes and cerebral infarction prognosis is important. Transient BP elevation is frequent in acute ischemic stroke and may increase perfusion of the ischemic penumbra (Miao et al., 2006). Hypoperfusion can lead to less perfusion and aggravate infarction (Olsen et al., 1983; Janardhan and Qureshi, 2004). Some of the hypertensive responses to acute ischemic stroke can result in a spontaneous BP decrease nearly to the former or normal level (Qureshi et al., 2007). Acute ischemic stroke may produce some stress factors that temporarily damage the BP regulation function (Murros et al., 1993). These factors can gradually diminish during vascular recanalization and reperfusion therapy with BP gradually decreasing to normal levels (Qureshi et al., 1999). Thus, a decrease in BP, to a certain extent, immediately after IVT may indicate vascular recanalization and brain tissue reperfusion, and these factors are relevant to good prognosis (Endo et al., 2013; Liu et al., 2016).

Liu et al. (2016) found that a sudden drop in SBP of 20 mmHg or greater between two continuous BP measurements within the first 2 h after IVT was associated with vascular recanalization and good outcomes. Most recanalizations induced by rt-PA occurred during the first hour after IVT and rarely presented after 2 h (Ribo et al., 2006). Our study showed that BP decrease within the first hour after IVT was associated with a favorable outcome, thus predicting prognosis faster. We did not find a significant association between outcomes and a BP decrease of over 20 mmHg, perhaps because we did not calculate the difference value between two continuous BP values. Nagaraja et al. (2014) observed that patients with a BP decrease of over 20 mmHg were associated with improved clinical symptoms and NIHSS scores. Mattle et al. (2005) also found that SBP within 24 h after IVT decreased significantly faster in patients with good outcomes than in patients with failed recanalization. In our study, we did not perform vascular recanalization evaluation for all patients because of a lack of patient cooperation, but by evaluating changes in BP, we found that a BP decrease was relevant to a good prognosis, while a significant BP increase was associated with poor outcomes.

BP fluctuation (variability) also influences the prognosis of IVT (Aslanyan et al., 2003). In this study, we found that small BP fluctuations with SD <10 mmHg correlated with a good 3-month prognosis. The extent of BP decrease during IVT was not significantly related to prognosis; however, the extent of BP increase was relative to the outcome. In addition to large BP fluctuations, obvious BP increase (≥10 mmHg) also predicted a poor prognosis. An obvious decrease in BP is relevant to recanalization, while a large BP increase may be associated with hemorrhage transformation. Ko et al. (2010) found that SBP variability within 72 h after IVT was related to hemorrhage transformation. Endo et al. (2013) measured BP eight times within the first 25 h after IVT and found that patients with higher BP variability had a higher risk of hemorrhage transformation and mortality after thrombolysis. Our result that BP variability after IVT is related to the 3-month outcome is consistent with those of previous studies (Stead et al., 2006; Kellert et al., 2012; Endo et al., 2013; Chang et al., 2018). Liu et al. (2015) found that SBP variability during the first 24 h after IVT is negatively associated with cerebral reperfusion and the 3-month neurological outcome. Results of ECASS-I showed that low SBP/DBP and small BP fluctuation increased chances of a good 3-month prognosis for cerebral infarction. Mei Yong (Yong and Kaste, 2008) analyzed BP data of 793 patients in the ECASS-II trial and found that high variability of SBP was associated with 7-day hemorrhage transformation and predictive of a poor outcome.

High baseline BP and ASPECTS are more likely to cause BP to decrease, while hypertension history is less likely to cause BP to decrease. BP elevation may compensate for the reperfusion of the penumbra. When the average BP varies between 60 and 150 mmHg, capillary capacity can be regulated by constriction and dilation according to BP changes to maintain steady cerebral perfusion (Paulson et al., 1990). Local ischemic brain tissue at occlusive cerebral vessels loses cerebral autoregulation; thus, cerebral perfusion largely depends on BP. Excessively high or low BP are risk factors for poor prognosis (Symon et al., 1973). Kellert et al. (2011) found that the relationship between baseline BP and prognosis after IVT was S-shaped. When SBP elevated slightly to the 146–160 mmHg range, every 1 mm BP elevation was associated with a 10% higher good-prognosis. A similar outcome was observed when DBP was 80–90 mmHg, which suggested that proper BP elevation was beneficial to the penumbra. Thus, proper elevated baseline BP may increase blood flow and lead to recanalization, which tends to decrease BP after IVT. The ASPECTS evaluates the infarction condition and reflects collateral compensation. High ASPECTS is relevant to good perfusion. Patients with a history of hypertension have vascular hyaline degeneration; thus, their collateral compensation is poor (Tikhonoff et al., 2009), and consequently, they have poor recanalization and low baseline BP.

There are some limitations to our study. First, we excluded patients treated with antihypertensive agents before IVT; thus, we cannot use these findings to guide their BP control. Since we need guidance for BP control in all IVT patients, trials including patients treated with antihypertensive agents are warranted. Second, the study is based out of a single center, and the sample size is limited. Multi-center prospective trials are needed to obtain more reliable results.

## Conclusion

Patients with a decreased and steady BP during thrombolysis were more likely to have a favorable outcome. Baseline CT ASPECTS, baseline NIHSS score, and hypertension history were independent factors related to BP change. These findings could help predict the prognosis of patients treated with IVT and suggest that BP control during IVT might improve clinical outcomes.

## Data Availability Statement

The raw data supporting the conclusions of this article will be made available by the authors, without undue reservation.

## Ethics Statement

The studies involving human participants were reviewed and approved by the Human Ethics and Research Ethic committees of the First Hospital of Jilin University. The patients/participants provided their written informed consent to participate in this study.

## Author Contributions

ZW and CW conceived and designed the study, acquired the data, and drafted and revised the manuscript. All authors analyzed and interpreted the data and critically revised the manuscript for important intellectual content. All authors contributed to the article and approved the submitted version.

## Conflict of Interest

The authors declare that the research was conducted in the absence of any commercial or financial relationships that could be construed as a potential conflict of interest.
