# Supplementary material for: Effects of Early Changes in Blood Pressure During Intravenous Thrombolysis on the Prognosis of Acute Ischemic Stroke Patients
Source: Front Aging Neurosci. 2020 Dec 4;12:601471. doi: 10.3389/fnagi.2020.601471 (PMC7746539; doi:10.3389/fnagi.2020.601471)
Supplement: Supplementary file 1 [file Table_1.docx]

Supplementary Material

# Supplementary Table

Supplementary Table 1. Relationship between baseline data and cerebral infarction prognosis.

| Factor | | | 3m mRS | | P |
| --- | --- | --- | --- | --- | --- |
|  |  |  | Favorable outcome(0-2) | Unfavorable outcome(3-6) |  |
|  |  |  | n=294 | n=164 |  |
| Age | | year | 59.49±11.86 | 61.59±11.76 | 0.069 |
| Male | | n(%) | 211(71.8) | 121(73.8) | 0.644 |
| Personal history | Smoking | n(%) | 185(62.9) | 97(59.1) | 0.425 |
|  | Drinking | n(%) | 139(47.3) | 79(48.2) | 0.855 |
| Medical History | Coronary artery disease | n(%) | 23(7.8) | 19(11.6) | 0.181 |
|  | Atrial fibrillation | n(%) | 27(9.2) | 19(11.6) | 0.412 |
|  | Cerebral infarction / TIA | n(%) | 33(11.2) | 26(15.9) | 0.156 |
|  | Hypertension | n(%) | 118(40.0) | 88(54.0) | 0.005* |
|  | Diabetes mellitus | n(%) | 41(13.9) | 21(12.8) | 0.732 |
| Baseline BP | | mmol/L | 150.78±19.44 | 153.91±22.27 | 0.118 |
| Baseline NIHSS score | |  | 8.25±5.57 | 13.51±7.42 | <0.001* |
| Baseline ASPECT score | |  | 8.65±1.82 | 8.13±2.00 | 0.005* |
| Lab tests | Blood glucose | mmol/L | 6.47±2.45 | 6.82±2.53 | 0.154 |
|  | RBC count | 10^12/L | 4.80±0.50 | 4.75±0.64 | 0.303 |
|  | PLT count | 10^9/L | 206.25±54.09 | 201.44±58.42 | 0.376 |
|  | Fibrinogen | g/L | 3.08±0.63 | 3.04±0.65 | 0.519 |
|  | Triglyceride | mmol/L | 1.74±1.32 | 1.70±1.64 | 0.790 |
|  | Total cholesterol | mmol/L | 4.66±1.04 | 4.80±1.12 | 0.171 |
|  | LDL | mmol/L | 2.85±0.71 | 2.87±0.79 | 0.765 |
|  | HDL | mmol/L | 1.24±0.32 | 1.36±0.41 | 0.012* |
| ONT | | min | 182.54±56.14 | 187.04±62.78 | 0.432 |
| DNT | | min | 63.46±24.59 | 67.34±32.60 | 0.185 |
| BP decrease | | n(%) | 204(69.4) | 98(59.8) | 0.037* |
| Hemorrhage transformation | | n(%) | 47(16.0) | 39(23.9) | 0.041* |

*denotes P＜0.05 for comparing between two groups. BP, blood pressure; NIHSS, National Institute of Health Stroke Scale; ASPECT, Alberta Stroke Program Early CT Score; TIA, transient ischemic attacks; RBC, red blood count; PLT, platelets; LDL, low density lipoprotein; HDL, high density lipoprotein; ONT, onset to needle time; DNT, door to needle time.
